# 1,25(OH) _2_D_3_ blocks IFNβ production through regulating STING in epithelial layer of oral lichen planus

**DOI:** 10.1111/jcmm.17409

**Published:** 2022-05-29

**Authors:** Xuejun Ge, Yaxian Wang, Hanting Xie, Ran Li, Fang Zhang, Bin Zhao, Jie Du

**Affiliations:** ^1^ Shanxi Province Key Laboratory of Oral Diseases Prevention and New Materials Shanxi Medical University School and Hospital of Stomatology Taiyuan Shanxi China; ^2^ Department of Endodontics Shanxi Medical University School and Hospital of Stomatology Taiyuan Shanxi China; ^3^ Department of Pathology Shanxi Medical University Taiyuan Shanxi China; ^4^ Department of Oral Medicine Shanxi Medical University School and Hospital of Stomatology Taiyuan Shanxi China; ^5^ Institute of Biomedical Research Shanxi Medical University Taiyuan Shanxi China

**Keywords:** GATA‐binding protein 1, interferon‐β, oral lichen planus, stimulator of interferon genes, vitamin D

## Abstract

Stimulator of interferon genes (STING) is reported to exert vital functions in inflammatory responses and autoimmune diseases. Nevertheless, the status and roles of STING in oral lichen planus (OLP) remain elusive. Here, we state that STING and its downstream cytokine interferon‐β (IFNβ) expression is boosted in the oral keratinocytes from patients suffering OLP in comparison with those from healthy participants. Mechanistically, transcription factor GATA‐binding protein 1 (GATA1) which is highly increased in diseased samples specifically interacts with its element in the promoter of *STING* to enhance STING transcripts. 1,25(OH)_2_D_3_, the active form of vitamin D, is capable of restricting STING and IFNβ increases in oral keratinocyte models resembling OLP in vitro. Moreover, there is a negative correlation between vitamin D receptor (VDR) and STING or IFNβ in human samples. Using plasmids and small interfering RNA transfection technologies, we find 1,25(OH)_2_D_3_ regulates STING and IFNβ through a mechanism controlled by the hypoxia‐inducible factor‐1α (HIF‐1α)‐GATA1 axis. Collectively, our findings unveil that 1,25(OH)_2_D_3_ lowers STING and IFNβ overexpression in the context of OLP.

## 1 INTRODUCTION

Oral lichen planus (OLP) is identified to be an inflammatory‐like mucocutaneous condition accompanied by recurrent lesions and symptoms.^1^ Recent systemic investigations have revealed that the prevalence of OLP in the globe is 1.01% and the risk for malignant transformation is approximately 1.2%.^2^, ^3^ OLP primarily affects the middle‐aged population, and the prevalence ratio between females and males is approximately 2:1.^2^ Although the pathogenic mechanism of OLP remains unclear, infectious, genetic, neurological, pharmacological, immunological and psychological contributors are all considered to exert critical functions in the pathogenesis of OLP.^4^ Based on recent publications, OLP is clinically classified as seven forms, among which reticular subtype is the most common in clinic.^1^ Most of OLP patients claim the symptoms like pain and a burning sensation in the diseased oral mucosa.^1^ Due to the shortage of curative management for this disease, exploration on the contributing factors of this illness is urgent.

Stimulator of interferon genes (STING), an adapter bound by cyclic GMP‐AMP (cGAMP), is located on the endoplasmic reticulum (ER) membrane of cells.^5^ STING expression is tightly modulated by transcriptional factors and posttranslational modifications as demonstrated.^6^ STING moves to the Golgi apparatus from the ER upon cGAMP binding, leading to IκB kinase (IKK) and TANK‐binding kinase 1 (TBK1) activation.^7^ These activated kinases induce downstream factors to increase pro‐inflammatory cytokine production, which mediates immune reactions.^7^, ^8^

Upregulation of STING is known to induce numerous autoimmunity and autoinflammatory diseases.^9^, ^10^, ^11^ In the sepsis animal model, compared to control wild‐type mice, sepsis severity is ameliorated in STING‐deficient mice.^12^, ^13^ Some investigations provide compelling evidence that STING insufficiency could decrease pro‐inflammatory cytokine production and arthritis scores in a self‐DNA‐mediated autoimmunity animal model.^14^ What is more, overexpression of STING mutation results in an autoinflammatory disease which shows a severe lung inflammation and acral vasculopathy.^15^

Vitamin D is reported to have critical roles in regulating autoimmune dysfunctions.^16^, ^17^, ^18^ In our previous explorations, we found that vitamin D concentration in the serum derived from OLP patients is decreased relative to that from healthy controls.^16^ In addition, we provided robust evidence that vitamin D/vitamin D receptor (VDR) pathway curbs cell apoptosis and pro‐inflammatory cytokine production in oral keratinocytes under inflammatory condition via regulating miRNAs, HIF‐1α signalling and renin levels.^19^, ^20^, ^21^, ^22^, ^23^, ^24^ However, the effect of vitamin D on STING and its downstream cytokines remains an enigma. Here, our data suggest 1,25(OH)_2_D_3_, the active form of vitamin D, decreases STING expression in oral keratinocyte to inhibit IFNβ production under OLP circumstance.

## 2 METHOD AND MATERIALS

### 2.1 Human biopsies

The diseased buccal tissues and blood samples were received from 14 OLP patients as described before.^16^ The normal mucosal and blood samples were obtained from 14 healthy individuals undergoing retained wisdom teeth extraction. Clinical OLP symptoms were identified in terms of the modified criteria. Clinical and function scores of OLP individuals were evaluated according to the previous investigation.^25^ Signed informed consents were received from participants. All human studies were approved by the Institutional Ethical Committee of Shanxi Medical University. Oral epithelial cells derived from human samples were isolated as previously described.^19^ Detailed information of human samples and statistical analysis between healthy controls and OLP groups were provided in Table S1–S3.

### 2.2 Cell culture

Cell line of human oral keratinocyte (HOK) was obtained and cultured in terms of previous investigation.^19^ Briefly, HOKs were cultured in oral keratinocyte medium (ScienCell) in the incubator under 37°C and 5% CO_2_ condition. Cell culture medium was replaced every 2 days. For mimicking OLP, 100 ng/ml lipopolysaccharide derived from Porphyromonas gingivalis (LPS‐PG, MilliporeSigma) or the supernatant from activated CD4^+^ T cells was separately used to stimulate HOKs for 8 h as described before.^19^ Anti‐human CD4 magnetic particles were applied for purifying CD4^+^ T cells from human peripheral blood samples. The enriched CD4^+^ T cells were further activated by anti‐CD3 and anti‐CD28 antibodies (BD Biosciences) and then cultured with RPMI 1640 medium. HOKs were pretreated with 1,25(OH)_2_D_3_ (20 nM) for 12 h prior to CD4^+^ T cells or LPS challenge. Plasmids or siRNAs were transfected into HOKs via vehicles for 36 h. 1,25(OH)_2_D_3_ was dissolved in ethanol, and the equivalent concentration of ethanol was used as negative control.

### 2.3 Western blot

Western blot was conducted as described before.^17^ Briefly, cell or tissue lysates were harvested with laemmli buffer with protease inhibitors. The same amounts of lysates were loaded and separated using SDS‐PAGE and then transferred onto a PVDF membrane. The membranes with proteins were blocked by 5% milk buffer, followed by an overnight primary antibodies' incubation. On Day 2, after TBST buffer washes, membranes were treated with HRP‐conjugated mouse or rabbit secondary antibodies at ambient temperature. Blots were visualized by using X‐ray films in dark room. Anti‐GATA‐binding protein 1 (GATA1) antibody was purchased from ProteinTech (Cat: 60011‐1‐Ig), anti‐STING antibody was purchased from ProteinTech (Cat:19851‐1‐AP), anti‐IFNβ antibody was purchased from ThermoFisher (Cat: PA5‐20390), and anti‐β‐actin antibody was purchased from Santa Cruz Biotechnology (Cat: sc‐47778).

Due to space limitations, more details were provided in supplemental materials (Appendix S1 and S2).

## 3 RESULTS

### 3.1 STING levels are upregulated in oral epithelial cells of OLP

**FIGURE 1:**
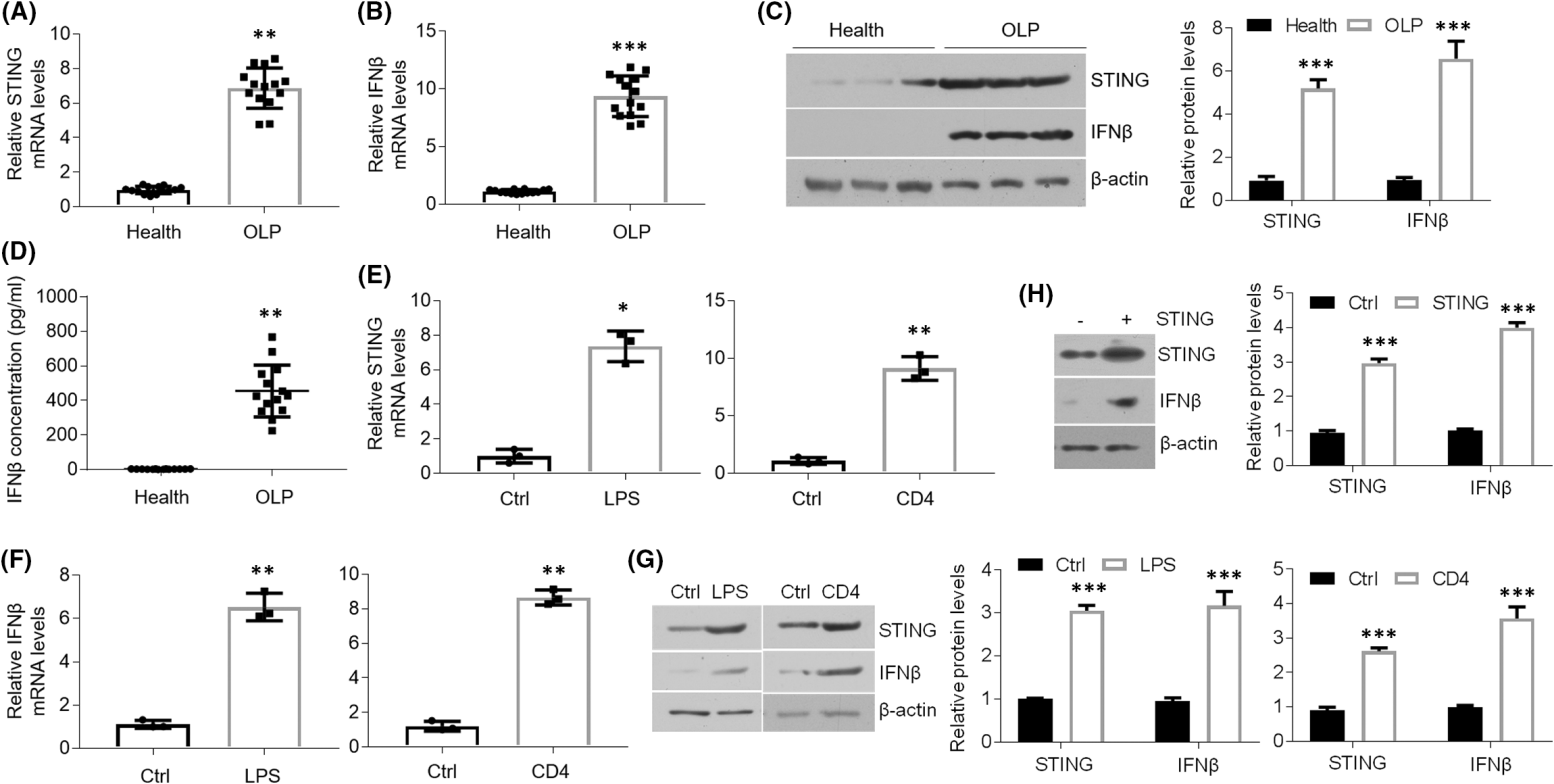
STING and IFNβ levels show increases in the unhealthy tissues from OLP. (A and B) Quantitative PCR (qPCR) analyses showing STING (A) and IFNβ (B) mRNAs expression in human biopsies. (C) STING and IFNβ protein status in human specimens measured by Western blot and densitometric quantitation. (D) Elisa measurement of IFNβ concentration in human serum. (E and F) qPCR analyses of STING (E) and IFNβ (F) mRNAs expression in HOKs treated with stimulators as noted. (G) Western blot detections and densitometric quantitation of STING and IFNβ protein levels in HOKs treated with stimulators as noted. (H) Western blot detections and densitometric quantitation showing STING and IFNβ protein status in HOKs in the presence or absence of STING plasmids transfection. *n* = 14 for human samples, *n* = 3 for HOK cell line. *p < 0.05, **p < 0.01 and ***p < 0.001 vs. corresponding control groups. All experiments were performed at least 3 times. Ctrl, control; LPS, lipopolysaccharide; OLP, oral lichen planus. Student's *t‐*test (A,B,D,E,F) was performed for statistical analysis

STING is reported to be closely associated with inflammatory diseases.^9^, ^10^, ^11^ To explore STING expression in OLP, we isolated oral mucosal epitheliums from healthy and OLP individuals for detection. As displayed, *STING* mRNA levels were remarkably elevated in oral epithelia derived from OLP patients compared to controls (Figure 1A). Accordantly, *IFNβ*, the downstream inflammatory factor of STING, was also increased in diseased human samples at the mRNA level (Figure 1B). Moreover, STING and IFNβ expression was also improved in lesion tissues at the protein level (Figure 1C). Likewise, serum IFNβ concentration showed a robust increase in OLP patients (Figure 1D). To test STING and IFNβ status in HOKs, we established two cell models for mimicking OLP as described before.^19^ As manifested, both mRNA and protein status of STING and IFNβ were enhanced in HOKs with challenges (Figure 1E‐G). Moreover, forced expression of STING increased IFNβ levels in HOKs (Figure 1H). These data above demonstrate that STING and its downstream gene levels are increased in oral keratinocytes in the setting of OLP.

### 3.2 STING is induced by GATA1 in oral keratinocytes

**FIGURE 2:**
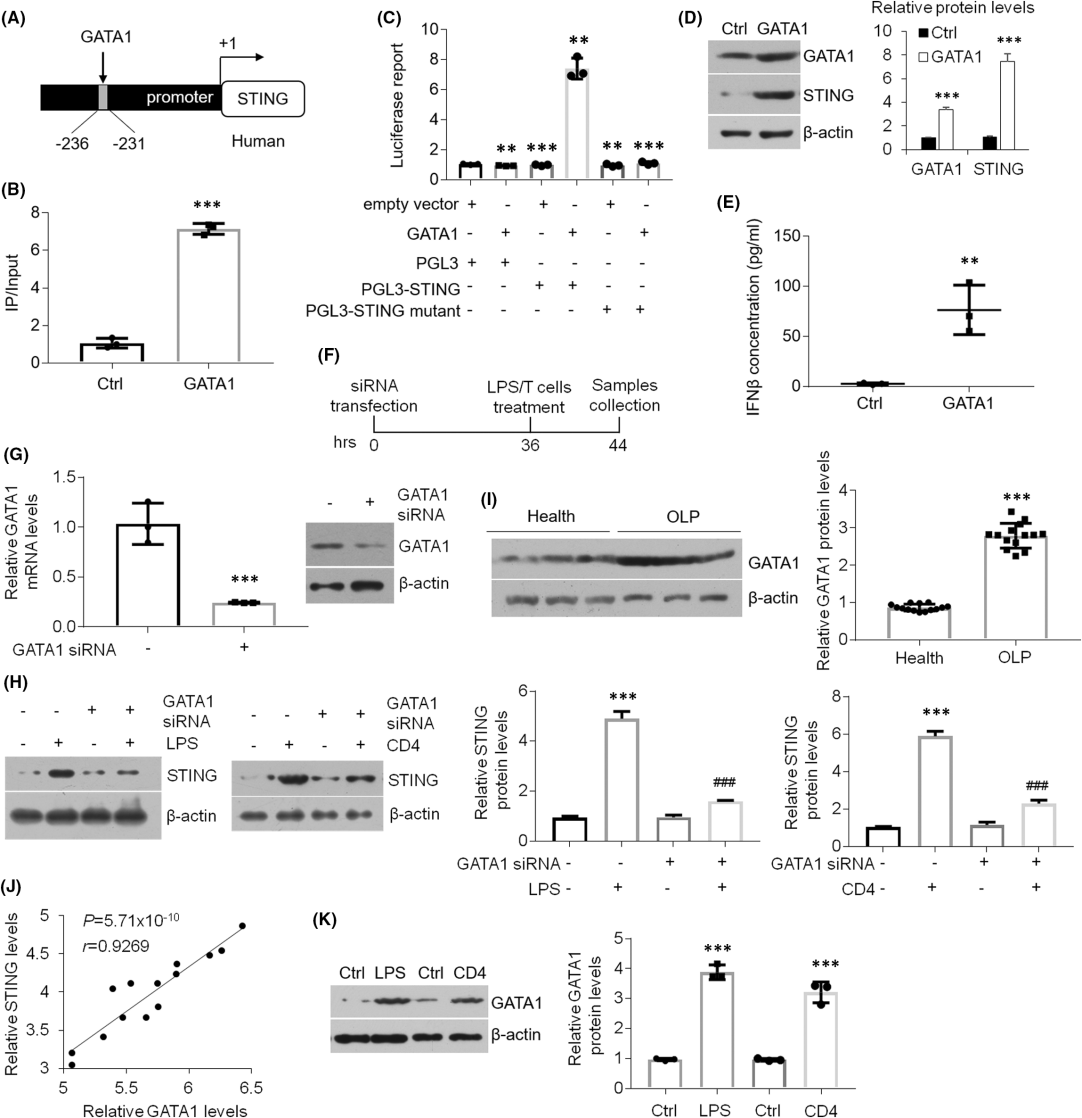
GATA1 induces *STING* transcripts in oral keratinocytes. (A) Schematic illustration for GATA1 motif in the promoter region of human *STING* gene. (B) ChIP assay against GATA1 or control antibody as indicated exhibiting the interaction of GATA1 with the element in the promoter of human *STING* gene in HOKs. (C) Luciferase report test showing HOKs transfected with GATA1 plasmids, PGL‐3 promoter vector harbouring the GATA1 element (PGL3‐STING) or PGL3‐STING mutant vector as indicated. (D) Western blot assays showing GATA1 and STING protein expression in GATA1 plasmids‐transfected HOKs. (E) IFNβ concentration in culture medium of HOKs transfected with GATA1 or empty vector tested by Elisa. (F) Schematic timing illustration of siRNA transfection and treatments. (G) qPCR and Western blot determinations of mRNA and protein levels in HOKs transfected with control or GATA1 siRNAs. (H) Western blot assays and densitometric quantitation showing STING protein status in stimulators‐treated HOKs with control or GATA1 siRNAs transfection. (I) Western blot detection and densitometric quantitation of GATA1 protein status in human samples. (J) Correlation analysis of STING and GATA1 protein levels in human samples. (K) Western blot analyses and densitometric quantitation of GATA1 levels in HOKs with treatments. *n* = 14 for human samples, *n* = 3 for HOK cell line. **p < 0.01 and ***p < 0.001 vs. corresponding control groups; ###*p* < 0.001 vs. LPS or CD4 group. All experiments were performed at least 3 times. Ctrl, control; LPS, lipopolysaccharide; OLP, oral lichen planus. Student's *t‐*test (B,E,G,J) and one‐way two‐sided ANOVA (C) were performed for statistical analysis

To explain the mechanism by which STING expression is boosted in the oral keratinocytes derived from OLP, we analysed the promoter area of *STING* and searched a potential GATA1‐binding site (Figure 2A). In addition, ChIP assays verified the binding action between transcript factor GATA1 and the element in the promoter of *STING* gene (Figure 2B). Luciferase report data confirmed transcript factor GATA1 promoted *STING* transcripts in HOKs transfected with PGL3‐STING plasmids but not the mutant ones. (Figure 2C). Overexpression of GATA1 in HOKs increased STING and IFNβ protein levels (Figure 2D,E). Next, we applied siRNA transfection in HOKs prior to challenges (Figure 2F). As manifested, knockdown of GATA1 in HOKs hampered STING increases upon stimulation (Figure 2G,H). Since GATA1 is closely associated with inflammatory responses,^26^, ^27^, ^28^ we investigated GATA1 expression in human biopsies and found GATA1 was increased in OLP samples compared to control ones (Figure 2I). Consistently, a positive correlation between STING and GATA1 was also observed in human specimens (Figure 2J). In OLP cell models, GATA1 status was raised by LPS or activated CD4^+^ T cells challenge (Figure 2K). Collectively, our results reveal that increased GATA1 in OLP induces STING expression by promoting its transcription.

### 3.3 1,25(OH)_2_D_3_ treatment decreases STING and IFNβ levels in oral keratinocytes of OLP

**FIGURE 3:**
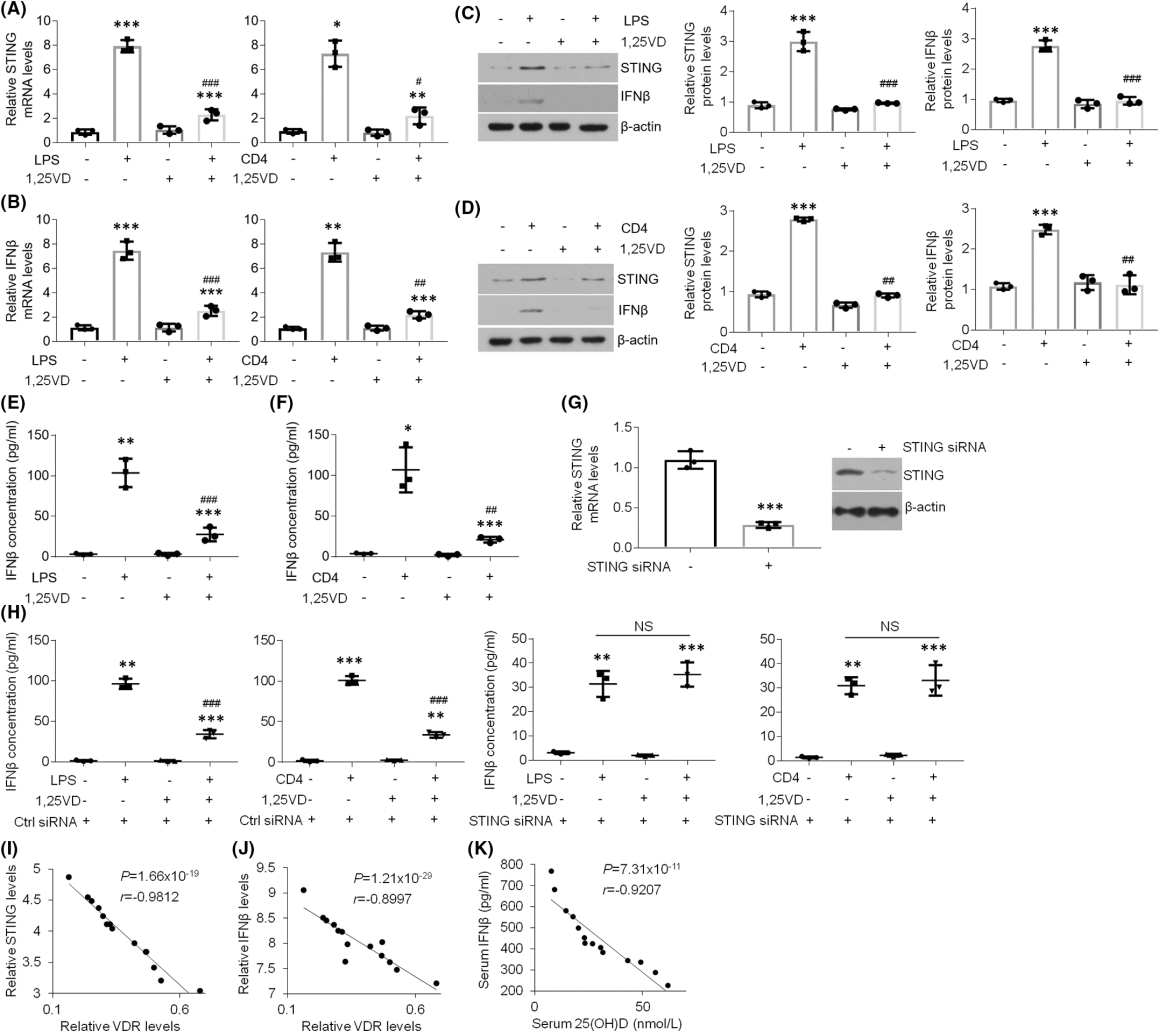
1,25(OH)_2_D_3_ suppresses STING expression. (A and B) qPCR analyses of STING (A) and IFNβ (B) mRNAs expression in HOKs challenged by LPS or CD4^+^ T cells with or without 1,25(OH)_2_D_3_. (C and D) Western blot tests and densitometric quantitation of STING and IFNβ protein status in HOKs challenged by LPS (C) or activated CD4^+^ T cells (D) with or without 1,25(OH)_2_D_3_. (E and F) Elisa measurements of IFNβ concentration in the culture medium of HOKs challenged by LPS (E) or activated CD4^+^ T cells (F) with or without 1,25(OH)_2_D_3_. (G) qPCR and Western blot determinations of mRNA and protein levels in HOKs transfected with control or STING siRNAs. (H) Elisa measurements of IFNβ concentration in the culture medium of control/STING siRNA‐transfected HOKs with various indicated treatments. (I and J) Correlations of STING and VDR (I), IFNβ and VDR (J) in human samples at the protein level. (K) Correlation of IFNβ and 25(OH)D in the serum of participants. *n* = 14 for human samples, *n* = 3 for HOK cell line. *p < 0.05, **p < 0.01 and ***p < 0.001 vs. corresponding control groups; #*p* < 0.05, ##*p* < 0.01 and ###*p* < 0.001 vs. corresponding LPS or CD4 group. All experiments were repeated at least 3 times. LPS, lipopolysaccharide; 1,25VD, 1,25(OH)_2_D_3_. Student's *t‐*test (G,I,J,K) and one‐way two‐sided ANOVA (A,B,E,F,H) were performed for statistical analysis

Although vitamin D is capable of regulating inflammatory reactions, its role in STING expression in the context of OLP remains elusive. To this end, we sought to elucidate whether vitamin D could mediate STING status in oral keratinocytes. As shown in Figure 3, treatment‐induced STING and IFNβ overexpression was suppressed by 1,25(OH)_2_D_3_ treatment in HOKs (Figure 3A‐D). The increased IFNβ concentrations in the culture medium of HOKs with challenges were also inhibited by 1,25(OH)_2_D_3_ (Figure 3E,F). Next, we knocked down STING expression in HOKs and indicated that 1,25(OH)_2_D_3_ refused to regulate IFNβ concentrations in LPS/activated CD4^+^ T cells‐treated HOKs with STING‐siRNAs transfection (Figure 3G,H), indicating vitamin D mediates IFNβ production via STING signalling. Moreover, after analysing the data of this project and previous investigation,^16^ we found VDR had negative correlations with STING and IFNβ in human oral samples (Figure 3I,J). The IFNβ and 25(OH)D concentrations in human serum showed a negative correlation as well (Figure 3K). These findings note that 1,25(OH)_2_D_3_ is capable of suppressing increased STING and IFNβ expression in the epithelia of OLP.

### 3.4 1,25(OH)_2_D_3_ is able to suppress GATA1 increases in oral keratinocytes

**FIGURE 4:**
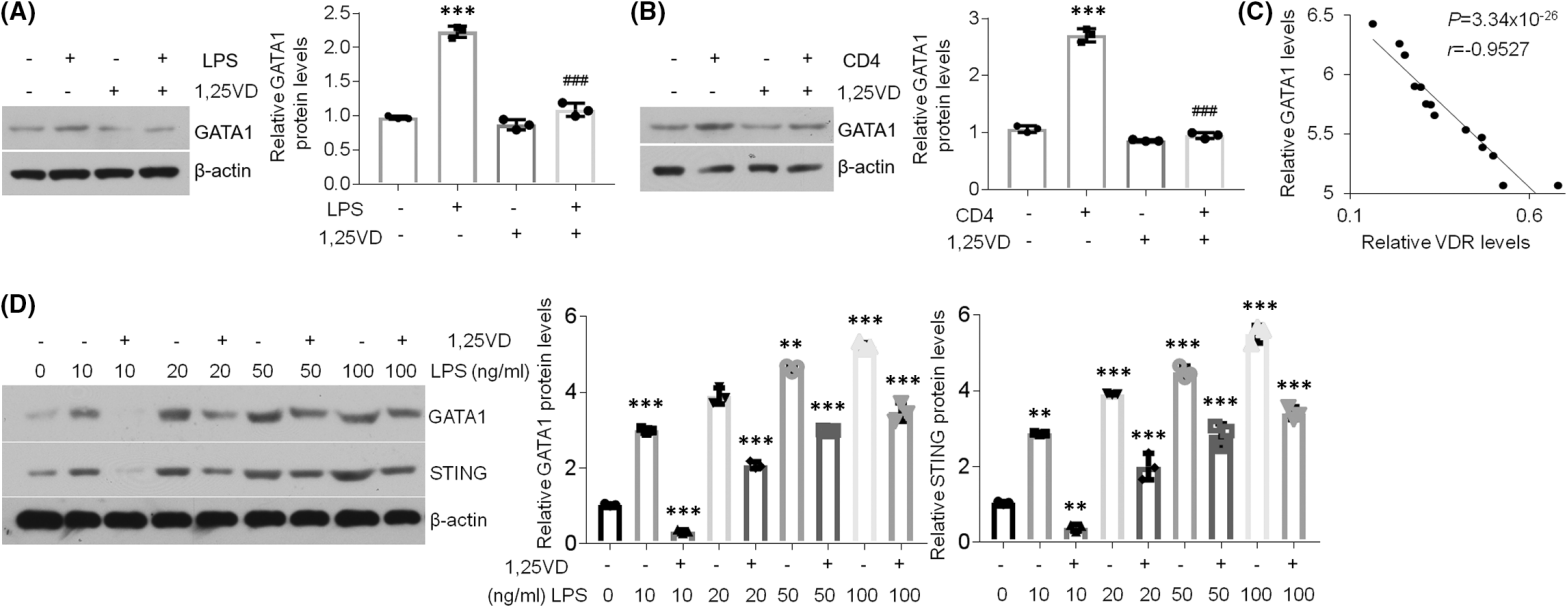
1,25(OH)_2_D_3_ inhibits GATA1 in oral keratinocytes. (A and B) Western blot tests and densitometric quantitation of GATA1 protein levels in HOKs challenged by LPS (A) or activated CD4^+^ T cells (B) with or without 1,25(OH)_2_D_3_. (C) Correlation of GATA1 and VDR in human samples at the protein level. (D) Western blot detections and quantitation of GATA1 and STING proteins levels in HOKs treated with various doses of LPS with or without 1,25(OH)_2_D_3_. *n* = 14 for human samples, *n* = 3 for HOK cell line. **p < 0.01 and ***p < 0.001 vs. corresponding control groups; ###*p* < 0.001 vs. LPS or CD4 group. All experiments were performed at least 3 times. LPS, lipopolysaccharide; 1,25VD, 1,25(OH)_2_D_3_. Student's *t‐*test (C) and one‐way two‐sided ANOVA (D) were performed for statistical analysis

Given that GATA1 can induce STING expression in oral keratinocytes, we next sought to better explain the functions of 1,25(OH)_2_D_3_ in GATA1. As exhibited, 1,25(OH)_2_D_3_ treatment stopped GATA1 elevations in HOKs upon stimulations (Figure 4A,B). Moreover, GATA1 and VDR showed a negative correlation in oral epithelia derived from human oral mucosa (Figure 4C). Finally, the dose‐dependent data indicated that 1,25(OH)_2_D_3_ failed to suppress STING expression when GATA1 levels were increasing in HOKs upon LPS treatment (Figure 4D), indicating 1,25(OH)_2_D_3_ blocks STING expression in a GATA1‐dependent way. Together, these data imply 1,25(OH)_2_D_3_ represses STING overexpression in oral keratinocytes via regulating GATA1 signalling.

### 3.5 1,25(OH)_2_D_3_ inhibits GATA1 levels by regulating HIF‐1α activation in oral keratinocytes

**FIGURE 5:**
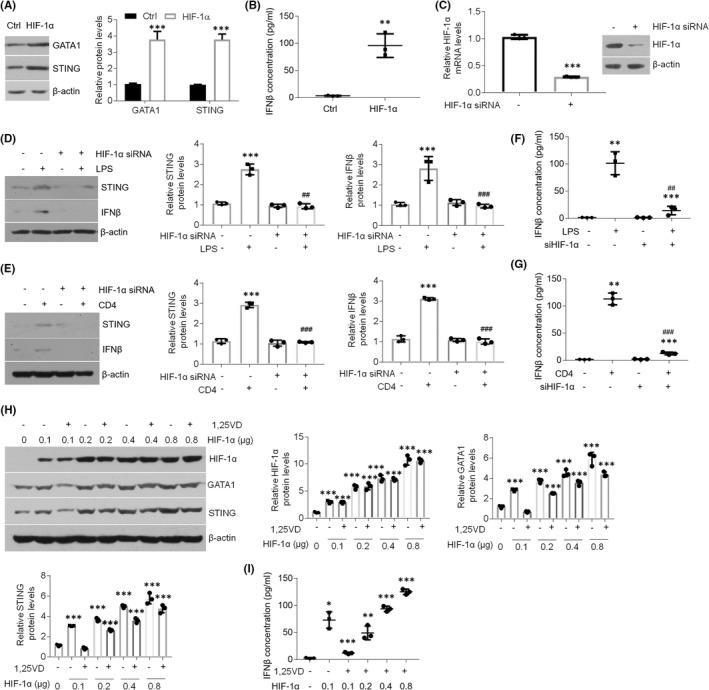
1,25(OH)_2_D_3_ regulates expression of HIF‐1α to suppress STING. (A) Western blot tests and densitometric quantitation showing GATA1 and STING protein status in HOKs transfected with or without HIF‐1α. (B) Elisa measurements of IFNβ concentration in the culture medium of HOKs with or without HIF‐1α transfection. (C) qPCR and Western blot determinations of mRNA and protein status in HOKs with or without control or HIF‐1α siRNAs transfection. (D and E) Western blot tests and densitometric quantitation of STING and IFNβ protein levels in scrambled or HIF‐1α siRNA‐transfected HOKs with or without LPS (D) or activated CD4^+^ T cells (E). (F and G) Elisa measurements of IFNβ concentration in the culture medium of scrambled or HIF‐1α siRNA‐transfected HOKs with or without LPS (F) or activated CD4^+^ T cells (G). (H) Western blot data and densitometric quantitation showing protein status in HOKs transfected with various doses of HIF‐1α plasmids with or without 1,25(OH)_2_D_3_. (I) Elisa evaluations of IFNβ concentration in the supernatants of HOKs transfected with various doses of HIF‐1α plasmids with or without 1,25(OH)_2_D_3_. *p < 0.05, **p < 0.01 and ***p < 0.001 vs. corresponding control groups; ##*p* < 0.01 and ###*p* < 0.001 vs. corresponding LPS or CD4 group. All experiments were performed at least 3 times. Ctrl, control; LPS, lipopolysaccharide; 1,25VD, 1,25(OH)_2_D_3_. Student's *t‐*test (B,C), one‐way two‐sided anova (F,G,I) were performed for statistical analysis

Some studies have suggested GATA1 is induced by HIF‐1α signalling.^29^, ^30^ To confirm this in oral keratinocytes, we transfected HIF‐1α plasmids into HOKs. Upon HIF‐1α overexpression, GATA1 expression was enhanced (Figure 5A), suggesting HIF‐1α signalling is able to induce GATA1 in HOKs. STING and IFNβ levels were also increased after HIF‐1α overexpression (Figure 5A,B). Meanwhile, LPS or activated CD4^+^ T cells lost the ability to increase STING and IFNβ when HIF‐1α was knocked down (Figure 5C‐G). In the dose‐dependent assay, 1,25(OH)_2_D_3_ suppressed HIF‐1α‐induced GATA1, STING and IFNβ upregulations when the dose of plasmids was 0.1 μg, but could not suppress them when HIF‐1α levels were increasing (Figure 5H,I), noting the indispensable role of HIF‐1α in vitamin D's mediation. These findings suggest 1,25(OH)_2_D_3_ suppresses GATA1 expression in oral keratinocytes dependent on HIF‐1α.

## 4 DISCUSSION

In this investigation, we tested human oral samples and found STING levels are significantly enhanced in diseased biopsies from OLP patients compared with those from healthy individuals, consistent with other studies indicating STING activation is elevated in autoimmune diseases such as colitis.^9^ Some explorations suggest that STING activation worsens experimental colitis in animals, leading to robust pro‐inflammatory cytokine production.^9^ Consistently, we confirmed that IFNβ and other inflammation‐related cytokine expression is promoted in the diseased tissues of OLP, indicating suppression of STING might be an efficient strategy to inhibit inflammatory responses in the context of OLP.

Given OLP is considered to be driven by T cells in the lamina propria,^1^ we collected T cells from humans and treated HOKs with the supernatants of activated CD4^+^ T cells to resemble the microenvironment of this inflammatory condition in vitro. To better mimic OLP, we also treated HOKs with LPS since bacterial infection is also reported to be a contributing factor for the initiation of OLP.^1^ Importantly, our HOKs and human samples data in this study are consistent.

Next, we explored the mechanism by which STING expression is upregulated. In line with other findings,^31^ we observed that GATA1 promotes *STING* transcription to enhance its status. Forced expression of GATA1 increases both STING and IFNβ in oral keratinocytes. GATA1 is closely related to inflammatory reactions, and conditional *GATA1* knockout in dendritic cells exhibits impaired migration towards lymph nodes.^26^ In addition, inflammasome of haematopoietic stem and progenitor cells mediates haematopoiesis via cleavage of GATA1.^27^ GATA1 also plays a critical role in inflammation‐associated thrombocytosis.^28^ Given the upregulated levels of GATA1 in diseased oral epithelium, targeting GATA1 in clinic might be a new tool for OLP therapy.

Calcitriol, a form of vitamin D, is indicated to suppress the cGAS/STING/IFN signalling in the field of Hutchinson Gilford Progeria Syndrome.^32^ Moreover, vitamin D exhibits the ability to dampen HIF‐1α expression through inactivating nuclear factor‐κB (NF‐κB) signalling pathway and enhancing von Hippel–Lindau (VHL) levels in oral keratinocytes.^20^ Other investigations suggest that vitamin D is able to suppress NF‐κB activity and inflammatory responses in human keratinocyte.^33^, ^34^ Accordingly, we found that 1,25(OH)_2_D_3_ is capable of repressing activated STING and IFNβ expression in the lesion tissues of OLP, suggesting vitamin D might ameliorate cytokine storm in oral epithelial cells by regulating STING signalling. One limitation is that cGAS part is not involved in the current work. Because there are no publications concerning cGAS and OLP so far, the study of them might be really interesting. In addition, we claimed that 1,25(OH)_2_D_3_ is able to suppress GATA1 to decrease STING expression in oral keratinocytes. Consistent with these findings that HIF‐1α induces GATA1 expression,^29^, ^30^ we provided compelling evidence that 1,25(OH)_2_D_3_ regulates GATA1 activation through HIF‐1α signalling in oral keratinocytes in this investigation.

In conclusion, this study suggests 1,25(OH)_2_D_3_ suppresses STING and its downstream cytokine IFNβ production by mediating GATA1 in the context of OLP, providing a novel target for the treatment of OLP. However, in addition to 25 and 1alpah hydroxylation, alternative pathways also could regulate vitamin D activation. For example, CYP11A1 triggers lumisterol (L3) metabolism to synthesize 20(OH)L3, 22(OH)L3 and 20,22(OH)_2_L3 via the side chain hydroxylation. Moreover, CYP11A1 exerts important functions in 7‐dehydrocholesterol (7DHC) which is closely associated with 7‐dehydropregnenolone (7DHP), 22(OH)_7_DHC and 20,22(OH)_2_7DHC production.^35^, ^36^ Based on recent investigations, AhR, liver X receptors (LXRs), RORα and RORγ are also found to be served as alternative receptors for vitamin D.^37^, ^38^, ^39^ Given the complicated biological network in the field of vitamin D, whether 1,25(OH)_2_D_3_ or targeting STING is qualified for OLP management requires more experimental and clinical trials.

## AUTHOR CONTRIBUTIONS

**Xuejun Ge:** Data curation (equal); investigation (lead). **Yaxian Wang:** Investigation (supporting). **Hanting Xie:** Investigation (supporting). **Ran Li:** Investigation (supporting); methodology (supporting). **Fang Zhang:** Investigation (supporting). **Bin Zhao:** Investigation (supporting). **Jie Du:** Conceptualization (lead); data curation (lead); formal analysis (lead); funding acquisition (lead); methodology (lead); project administration (lead); writing – original draft (lead); writing – review and editing (lead).

## CONFLICT OF INTEREST

There are no conflicts of interest in this project.

## Supporting information


Appendix S1

Appendix S2

## Data Availability

The data are available from corresponding author.
