# Supplementary material for: 1,25(OH) 2D3 blocks IFNβ production through regulating STING in epithelial layer of oral lichen planus
Source: J Cell Mol Med. 2022 May 29;26(13):3751–9. doi: 10.1111/jcmm.17409 (PMC9258715; doi:10.1111/jcmm.17409)
Supplement: Supplementary file 1 — Appendix S1 [file JCMM-26-3751-s002.docx]

**1,25(OH)_2_D_3_ blocks IFNβ production through regulating STING in epithelial layer of oral lichen planus**

Xuejun Ge^1,2^, Yaxian Wang^1,2^, Hanting Xie^1,3^, Ran Li^1^, Fang Zhang^1,4^, Bin Zhao^1^, Jie Du^1,4,5*^

^1^Shanxi Province Key Laboratory of Oral Diseases Prevention and New Materials, Shanxi Medical University School and Hospital of Stomatology, Taiyuan, Shanxi, China.

^2^Department of Endodontics, Shanxi Medical University School and Hospital of Stomatology, Taiyuan, Shanxi, China.

^3^Department of Pathology, Shanxi Medical University, Taiyuan, Shanxi, China.

^4^Department of Oral Medicine, Shanxi Medical University School and Hospital of Stomatology, Taiyuan, Shanxi, China.

^5^Institute of Biomedical Research, Shanxi Medical University, Taiyuan, Shanxi, China.

*Corresponding author: Jie Du, D.D.S., Ph.D., Shanxi Province Key Laboratory of Oral Diseases Prevention and New Materials, Shanxi Medical University School and Hospital of Stomatology, Taiyuan, Shanxi, 030001, China (email: [dj1243@hotmail.com](mailto:dj1243@hotmail.com)).

Supplemental Tables

Supplemental Table 1. Information of healthy individuals involved in this study

| NO. | Age | Gender | Sites |
| --- | --- | --- | --- |
| 1 | 23 | F | Buccal mucosa |
| 2 | 24 | F | Buccal mucosa |
| 3 | 36 | M | Buccal mucosa |
| 4 | 19 | F | Buccal mucosa |
| 5 | 20 | F | Buccal mucosa |
| 6 | 27 | F | Buccal mucosa |
| 7 | 32 | M | Buccal mucosa |
| 8 | 18 | M | Buccal mucosa |
| 9 | 26 | M | Buccal mucosa |
| 10 | 28 | M | Buccal mucosa |
| 11 | 24 | F | Buccal mucosa |
| 12 | 22 | M | Buccal mucosa |
| 13 | 20 | M | Buccal mucosa |
| 14 | 29 | F | Buccal mucosa |

Supplemental Table 2. Demographic and clinical characteristics of the patients and healthy controls

| Clinical characteristics | n | Healthy control (n = 14) | OLP  (n = 14) | Yates' *P*-value |
| --- | --- | --- | --- | --- |
| Age (years) | | | | |
| <30 | 13 | 12 | 1 | 0.0011 |
| 30-50 | 9 | 2 | 7 |  |
| >50 | 6 | 0 | 6 |  |
| Gender | | | | |
| Male | 11 | 7 | 4 | 0.4390 |
| Female | 17 | 7 | 10 |  |
| Clinical presentation | | | | |
| Reticular | 12 | 0 | 12 | 0.7024 |
| Reticular and Plaque | 2 | 0 | 2 |  |
| Site of involvement | | | | |
| Buccal mucosa | 26 | 14 | 12 | 0.4633 |
| Buccal mucosa and Tongue | 2 | 0 | 2 |  |
| Serum 25(OH)D concentration (nmol/L) | | | | |
| <50 | 12 | 0 | 12 | 0.0003 |
| 50-100 | 12 | 10 | 2 |  |
| >100 | 4 | 4 | 0 |  |

*The chi-square test with Yates’ correction was applied for the statistical analysis here.

Supplemental Table 3. Correlation between STING expression of oral epithelia and clinical characteristics in OLP patients

| Clinical characteristics | n | STING expression  High (n = 8) | STING expression  Low (n = 6) | Yates' *P*-value |
| --- | --- | --- | --- | --- |
| Age (years) | | | | |
| <30 | 1 | 0 | 1 | 0.9185 |
| 30-50 | 7 | 4 | 3 |  |
| >50 | 6 | 4 | 2 |  |
| Gender | | | | |
| Male | 4 | 3 | 1 | 0.7973 |
| Female | 10 | 5 | 5 |  |
| Clinical presentation | | | | |
| Reticular | 12 | 7 | 5 | 0.5814 |
| Reticular and Plaque | 2 | 1 | 1 |  |
| Site of involvement | | | | |
| Buccal mucosa | 12 | 7 | 5 | 0.5814 |
| Buccal mucosa and Tongue | 2 | 1 | 1 |  |
| Clinical scores | | | | |
| 1 | 4 | 1 | 3 | 0.2300 |
| 2 | 5 | 2 | 3 |  |
| 3 | 5 | 5 | 0 |  |
| Clinical function scores | | | | |
| 1 | 1 | 0 | 1 | 0.2274 |
| 2 | 3 | 0 | 3 |  |
| 3 | 5 | 3 | 2 |  |
| 4 | 5 | 5 | 0 |  |

* The chi-square test with Yates’ correction was applied for the statistical analysis here.

Supplemental Table 4. Primers involved in this study.

| Primer name | Forward(5′-3′) | Reverse(5′-3′) |
| --- | --- | --- |
| hHIF-1α | GAACGTCGAAAAGAAAAGTCTCG | CCTTATCAAGATGCGAACTCACA |
| hIFNβ | ATGACCAACAAGTGTCTCCTCC | GGAATCCAAGCAAGTTGTAGCTC |
| hSTING | CCAGAGCACACTCTCCGGTA | CGCATTTGGGAGGGAGTAGTA |
| hGATA1 | CTGTCCCCAATAGTGCTTATGG | GAATAGGCTGCTGAATTGAGGG |
| hGAPDH | ACCACAGTCCATGCCATCAC | TCCACCACCCTGTTGCTGTA |
| STING ChIP | TGGCTGAGGAGCTGCGCCA | CCCGTCCCGATCCGTAGT |
| hGATA1 cDNA | ATGGAGTTCCCTGGCCTGGGGT | TCGGTCCCCCACGGACCCCG |
| hSTING  cDNA | ATGCCCCACTCCAGCCTGCA | AGTTCTCTTTAGGCACGCCT |

**Method and materials**

RT–PCR. The whole RNAs were isolated with TRIzol reagent (Invitrogen). For mRNAs analyses, 500 ng RNAs were generated into cDNA by a commercial RT kit (TOYOBO). Real-time PCR examinations were then adopted for quantitative analysis using a commercial qPCR kit (TOYOBO) and a real-time PCR system (Roche). GAPDH was selected to be the endogenous control. Related primers are listed in supplemental table 4.

Elisa. IFNβ concentrations in the human serum and cell culture media were determined by a purchased ELISA kit (PBL Assay Science, Cat: 41410) in terms of the manufacturer’s instructions.

Chromatin immunoprecipitation (ChIP) assays. HOKs were fixed with formaldehyde (1%), followed by glycine (0.125 M) treatment at ambient temperature. After two washes with cold PBS, fixed cells were re-dissolved in lysis buffer. The cell lysates were sonicated to get approximately 500 bp chromatin fragments. 10% sonicated lysates were saved for INPUT, the rest was rotated with anti-GATA1 antibody or IgG monoclonal antibody at 4 °C for 12 h prior to protein A agarose beads supplements. After washes with high salt buffer, low-salt buffer and TE buffer, these beads were eluted and DNA was purified from the supernatant. All samples were quantified by qPCR. INPUT was used for internal controls. Related primers are listed in supplemental table 4.

Plasmids construction. For plasmids generation, the coding sequences of human *GATA1* or *STING* cDNA were amplified and subcloned into pcDNA3.1 vector to construct GATA1 or STING plasmids. For luciferase reporter plasmid constructions, 50 bp DNA sequences (ACTCACTGCAGTACCCAGGGACGGGGTATCCAA CGTGTGTCACTCCCTTG) containing potential GATA1 binding site in the promoter region of human *STING* were synthesized and inserted into a PGL3 vector. Mutated sequences (ACTCACTGCAGTACCCAGGGACGGGGTAAAAAACGTGTGTCACTCCCTTG) containing the potential binding site were synthesized with a Site-Directed Mutagenesis Kit (Agilent).

Transfection assay. Plasmids (4 µg) and siRNA oligonucleotides (40 µM) were transfected into oral keratinocytes transiently by using Lipofectamin 3000 (Invitrogen). The target sequence of hHIF-1α-siRNA was 5′-AGAGGUGGAUAUGUGUGGGdTdT-3′. The siRNA sequences (sense) of GATA1 and STING are: 5′-AGUUGAGGCAGGGUAGAGCtt-3′ and 5′-UCAUAAACUUUGGAUGCUA-3′, respectively. Scramble siRNAs were served as controls.

Luciferase activity detection. HOKs were transfected with PGL3-STING, PGL3-mutant or control plasmids for 36 hrs. Luciferase activity was detected by the Dual Luciferase Reporter Assay System (Promega, cat: E1910) in terms of manufacturer’s instructions.

Statistical analysis. Data values were exhibited as means ± SD. Unpaired two‐tailed Student’s *t* test was used for two groups’ comparisons, and one‐way analysis of variance (ANOVA) was for three or more groups’ comparisons. The chi-square test was applied to analyze demographic and clinical characteristics of human samples. *P* < 0.05 was recognized to be statistically significant.
