# Supplementary figures and images for: 1,25(OH) 2D3 blocks IFNβ production through regulating STING in epithelial layer of oral lichen planus
Source: J Cell Mol Med. 2022 May 29;26(13):3751–9. doi: 10.1111/jcmm.17409 (PMC9258715; doi:10.1111/jcmm.17409)

Raw data


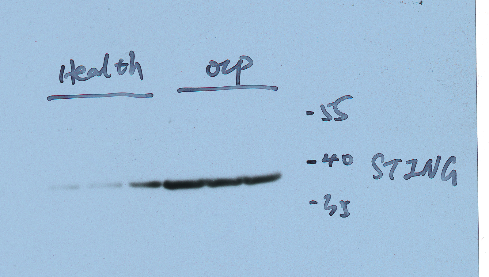

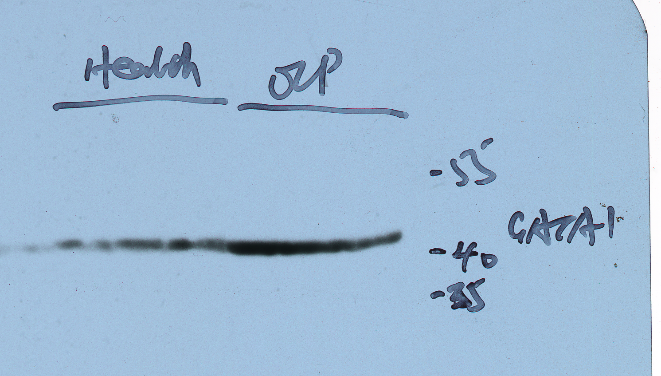


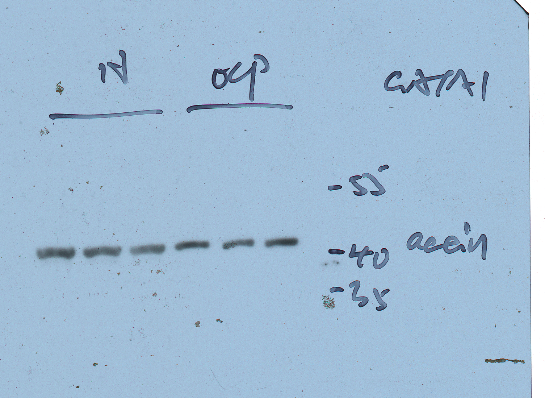

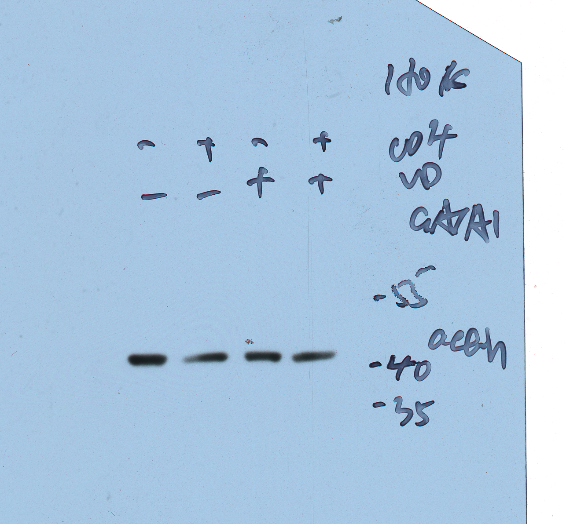

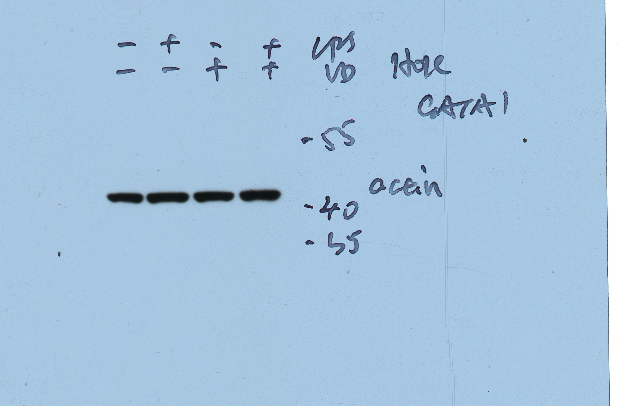

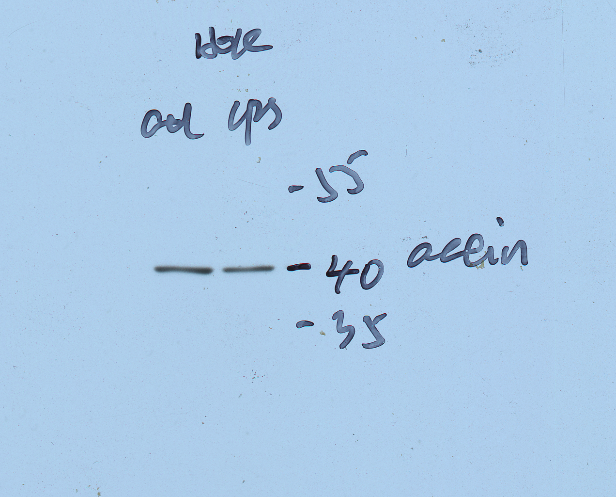

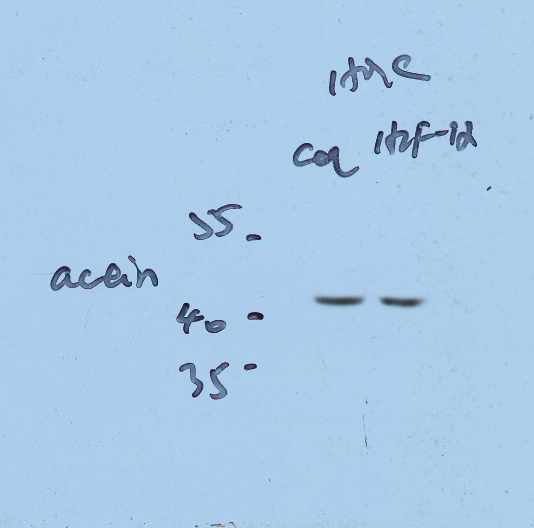

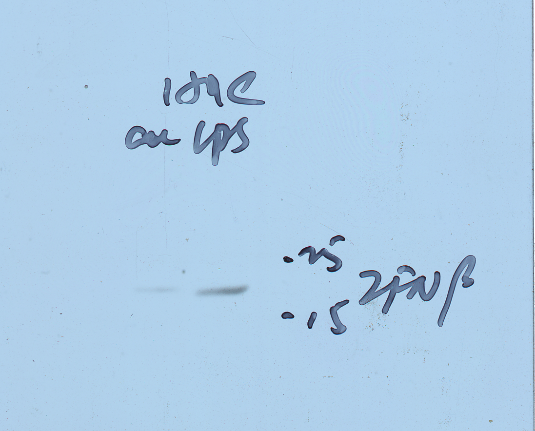

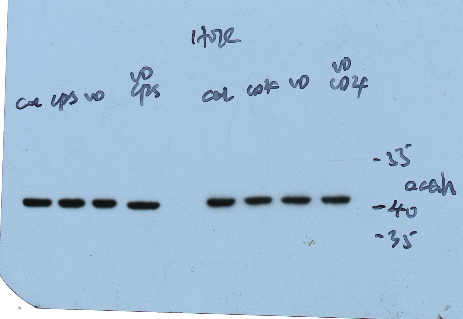

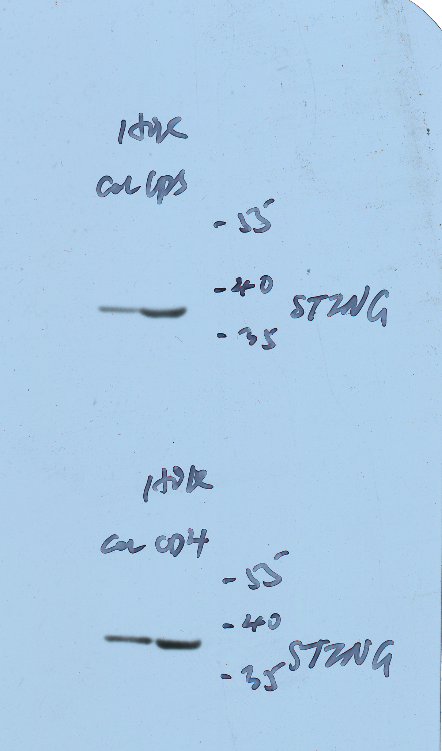

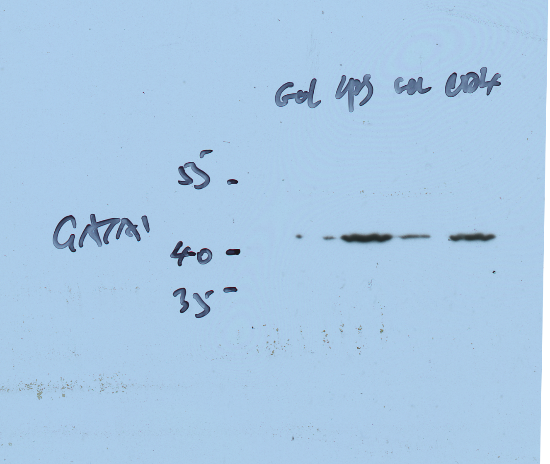

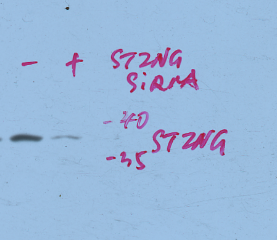

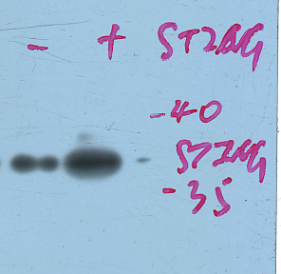

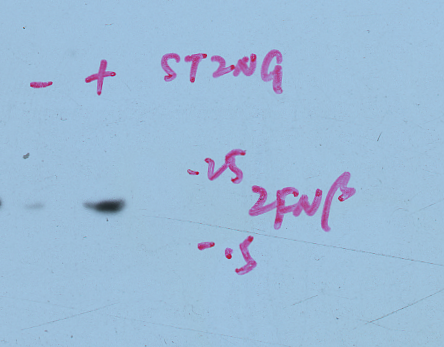

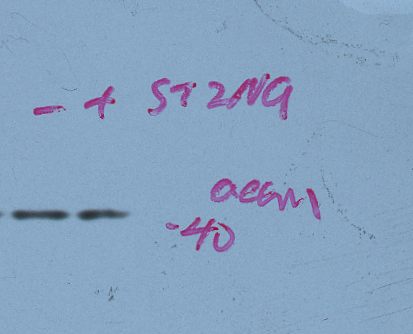

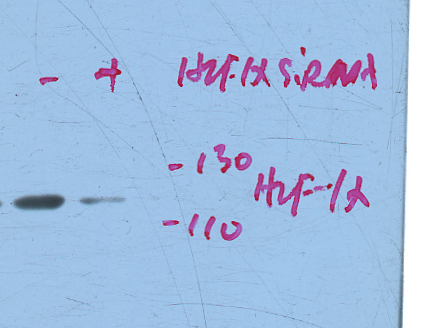

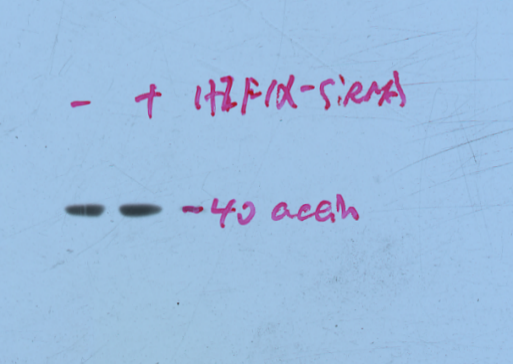

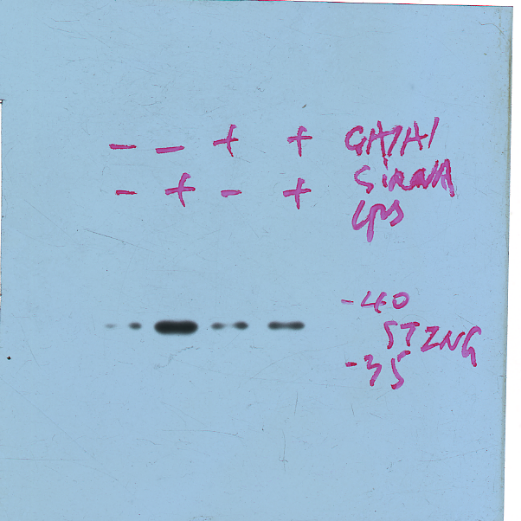

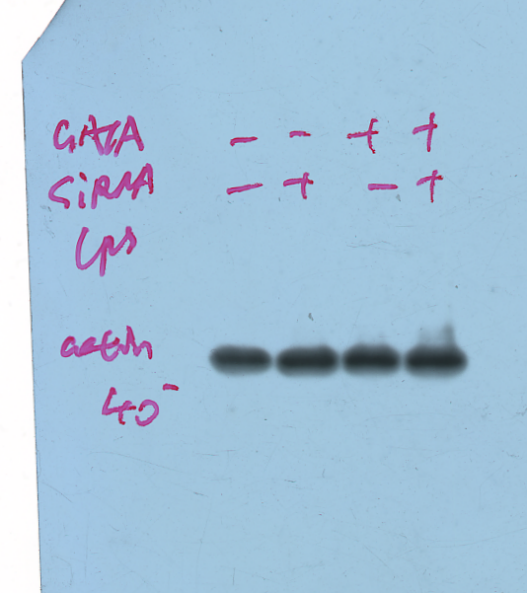

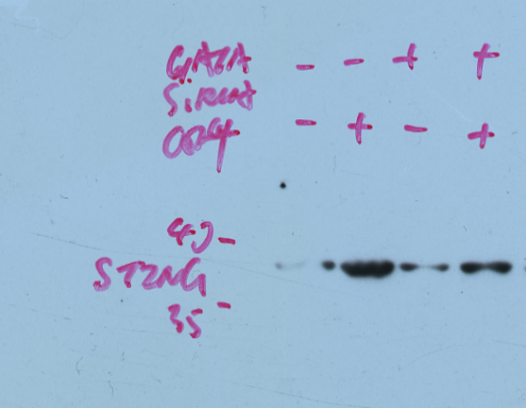

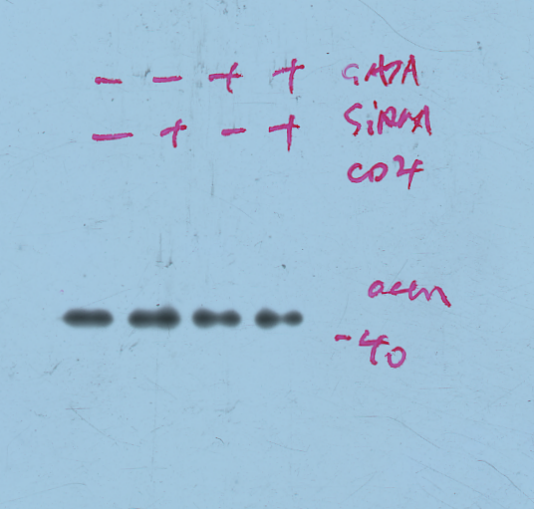


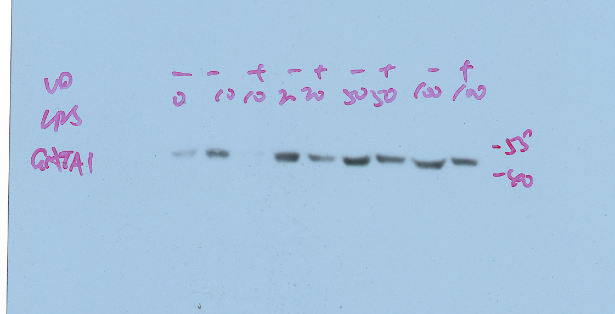

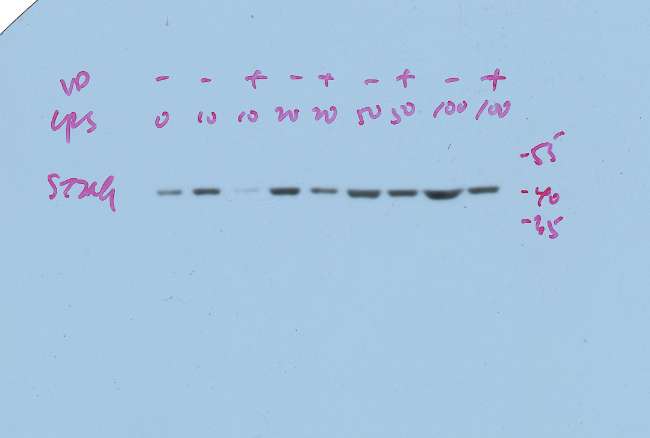

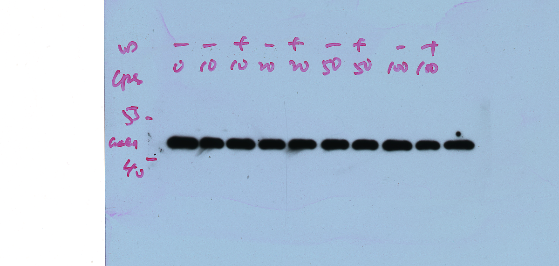

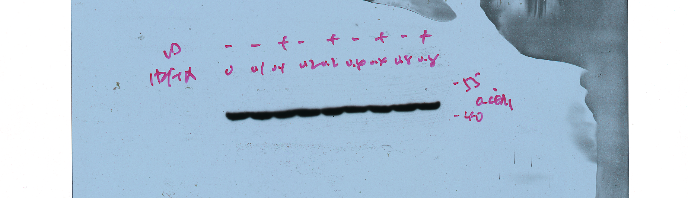

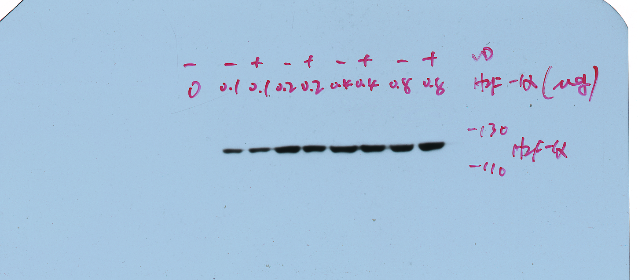

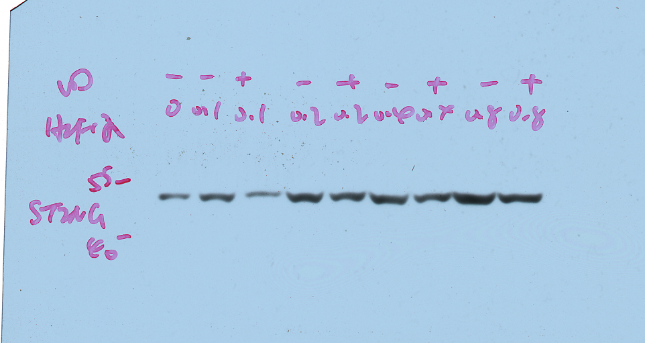


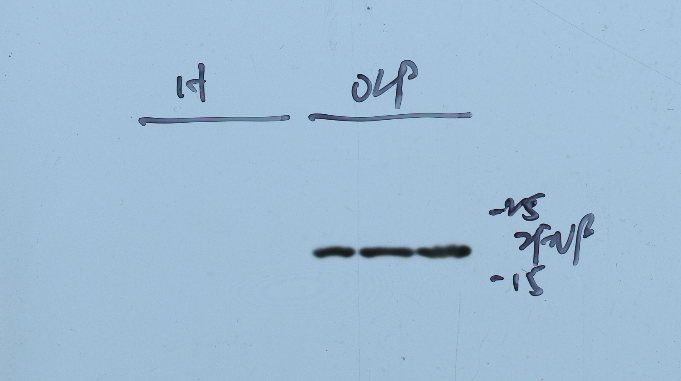

Supplement: Supplementary file 2 — Appendix S2 [file JCMM-26-3751-s001.docx]
